# Engineering protein glycosylation in CHO cells to be highly similar to murine host cells

**DOI:** 10.3389/fbioe.2023.1113994

**Published:** 2023-02-16

**Authors:** Shivani Gupta, Bhavana Shah, Coral Shek Fung, Pik Kay Chan, Devin L. Wakefield, Scott Kuhns, Chetan T. Goudar, James M. Piret

**Affiliations:** ^1^ Amgen, Inc., San Francisco, CA, United States; ^2^ Michael Smith Laboratories, and Department of Chemical and Biological Engineering, University of British Columbia, Vancouver, BC, Canada; ^3^ Amgen, Inc., Thousand Oaks, CA, United States; ^4^ Amgen, Inc., Cambridge, MA, United States

**Keywords:** biosimilar, glycosylation, cell line engineering, Chinese Hamster ovary (CHO), murine

## Abstract

Since 2015 more than 34 biosimilars have been approved by the FDA. This new era of biosimilar competition has stimulated renewed technology development focused on therapeutic protein or biologic manufacturing. One challenge in biosimilar development is the genetic differences in the host cell lines used to manufacture the biologics. For example, many biologics approved between 1994 and 2011 were expressed in murine NS0 and SP2/0 cell lines. Chinese Hamster ovary (CHO) cells, however, have since become the preferred hosts for production due to their increased productivity, ease of use, and stability. Differences between murine and hamster glycosylation have been identified in biologics produced using murine and CHO cells. In the case of monoclonal antibodies (mAbs), glycan structure can significantly affect critical antibody effector function, binding activity, stability, efficacy, and *in vivo* half-life. In an attempt to leverage the intrinsic advantages of the CHO expression system and match the reference biologic murine glycosylation, we engineered a CHO cell expressing an antibody that was originally produced in a murine cell line to produce murine-like glycans. Specifically, we overexpressed cytidine monophospho-N-acetylneuraminic acid hydroxylase (CMAH) and N-acetyllactosaminide alpha-1,3-galactosyltransferase (GGTA) to obtain glycans with N-glycolylneuraminic acid (Neu5Gc) and galactose-α-1,3-galactose (alpha gal). The resulting CHO cells were shown to produce mAbs with murine glycans, and they were then analyzed by the spectrum of analytical methods typically used to demonstrate analytical similarity as a part of demonstrating biosimilarity. This included high-resolution mass spectrometry, biochemical, as well as cell-based assays. Through selection and optimization in fed-batch cultures, two CHO cell clones were identified with similar growth and productivity criteria to the original cell line. They maintained stable production for 65 population doubling times while matching the glycosylation profile and function of the reference product expressed in murine cells. This study demonstrates the feasibility of engineering CHO cells to express mAbs with murine glycans to facilitate the development of biosimilars that are highly similar to marketed reference products expressed in murine cells. Furthermore, this technology can potentially reduce the residual uncertainty regarding biosimilarity, resulting in a higher probability of regulatory approval and potentially reduced costs and time in development.

## 1 Introduction

Recombinant therapeutic proteins, also known as biologics, have been successfully used to treat a wide range of diseases, including cancers, inflammatory disorders, and cardiovascular diseases (Walsh, 2018). Biosimilars with comparable efficacy and safety profiles have emerged as alternative options to innovator therapies. A biosimilar is a biologic that is highly similar to an originator biologic drug (“reference biologic” or RB) with no clinically meaningful differences from the RB. Regulations in the United States and European Union provide for abbreviated approval pathways for biosimilars, and the United States Food and Drug Administration (FDA) (FDA, 2020) and European Medicines Agency (EMA) (Jung et al., 2020) have issued guidelines further describing regulatory expectations for biosimilars development (FDA, 2020).

Successful development of a biosimilar requires achieving product characteristics that are similar to the characteristics of the RB. In particular, protein glycosylation, the attachment of carbohydrates to a protein structure, can significantly impact binding affinity, immune effector functions, stability, and safety (Cymer et al., 2018). Thus, a critical component of biosimilar development is achieving a glycan profile that is highly similar to that of the RB. In many cases, optimization of the culture medium, such as adding glycan precursors, can modulate glycosylation (Naik et al., 2018; Bruhlmann et al., 2017). However, achieving the similarity of the complex and varied post-translational modifications can be challenging, especially when different production cells and manufacturing processes are used for a biosimilar compared to the RB. Of note, Chinese Hamster ovary (CHO) cells are currently the host cells of choice industry-wide because of generally well-established procedures resulting in robust and stable production of biologics and because of the long-established history of regulatory approval for producing safe and efficacious biologics (Wang et al., 2018).

**FIGURE 1:**
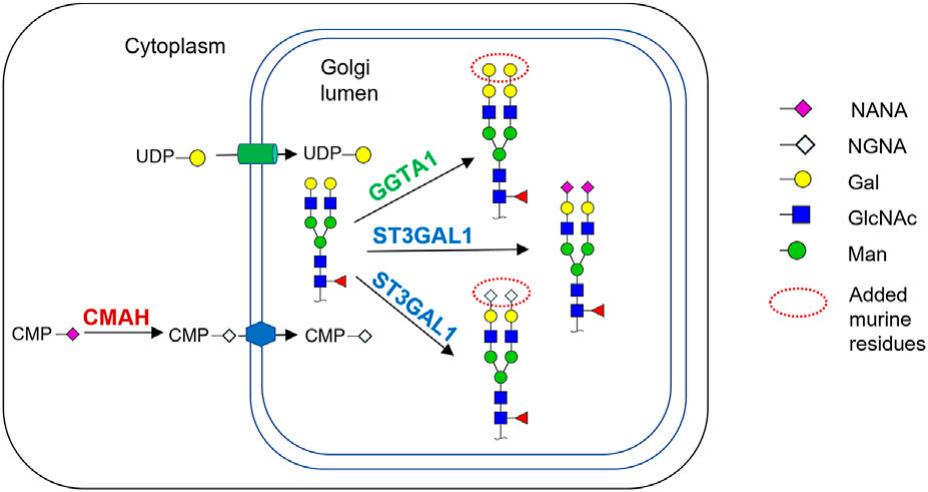
Schematic of the relevant parts of the N-glycosylation pathway in murine myeloma cell lines and the CHO cells engineered in this work. Cytidine-5′-monophospho-N-Glycolylneuraminic acid (CMP-NGNA) is synthesized in the cytosol by the CMP-Neu5Ac hydroxylase (CMAH) hydroxylation of the N-acetyl group of Cytidine-5′-monophospho-N-acetylneuraminic acid (CMP-NANA). In the Golgi, CMP-NGNA is transferred to galactose-containing glycan substrates by sialyltransferases denoted by ST. The Golgi glycosylation enzyme GGTA1 transfers galactose from UDP-gal to the terminal galactose residue, producing the α-Gal glycan. In this work, the CHO cells were genetically engineered to express CMAH and GGTA1.

Increasingly, mechanistic understanding of the repertoire of glycosyltransferases and other glycan modifying enzymes have guided cell-line engineering to tailor the glycan profiles of biologics (Wang et al., 2018). Here we investigated the production of a biosimilar candidate, a monoclonal antibody (“mAb”), for an approved recombinant human-mouse chimeric originator biologic produced SP2/0 cell line. This RB contains a conserved glycan fragment on both crystallizable fragment (Fc) and antigen-binding (Fab) regions. Notably, murine SP2/0 cells express N-acetyllactosaminide alpha-1,3-galactosyltransferase (GGTA1) and cytidine monophospho-N-acetylneuraminic acid hydroxylase (CMAH), which are responsible for the presence of α-Gal and NGNA, respectively (Figure 1). However, genes encoding these two enzymes are absent in CHO cells (Goh and Ng, 2018). Human cells do not produce glycans with α-Gal and NGNA epitopes. Therefore, α-Gal and NGNA can elicit an immunogenic response in humans (Jenkins et al., 1996; Raju et al., 2000; Baker et al., 2001; Bosques et al., 2010). In addition, SP2/0 cells express both α-2,3-sialyltransferase (ST3GAL1) and α-2,6-sialyltransferase (ST6GAL1), the critical enzymes involved in the addition of the sialic group, N-acetyl-neuraminic acid (NANA or NGNA) to the mAb. In contrast, CHO cells lack functional ST6GAL1 and CMAH and, therefore, exclusively produce α (2,3)-linked NANA (Goh and Ng, 2018). Hence, to produce a glycan profile in CHO cells similar to that of the RB, it was essential to compensate for differences in glycosylation enzymes between the murine and CHO cell lines.

The main hypothesis of this work was that CHO cells can be genetically engineered to express murine cell glycosylation in a recombinant antibody protein while maintaining the cell line production stability and product critical quality attributes required for biosimilar manufacturing. To investigate this goal, a CHO cell line expressing the RB mAb was engineered to produce murine-like glycans. Specifically, CMAH and GGTA1 were overexpressed to obtain glycans with N-glycolylneuraminic acid (Neu5Gc; NGNA) and galactose-α-1,3-galactose (alpha gal; α-Gal). A diverse set of clones was then screened to find stable CHO production cell lines. These were tested in fed-batch cultures to determine their production performance. Then a wide range of assays was performed to determine whether the desired complex critical quality attributes (CQAs) of the target biosimilar candidate were maintained.

## 2 Methods

### 2.1 Media and reagents

Company-proprietary vector and media (Amgen Inc., Thousand Oaks, CA, United States) were used throughout the study unless otherwise specified. An Amgen generated dihydrofolate reductase (DHFR) deficient CHO cell line expressing mAb with the same amino acid sequence as RB.

### 2.2 Design of vectors for CHO cell line engineering

Gene fragments for the mouse genes encoding CMAH and GGTA1, the glycosylation enzymes were synthesized (Geneart, Thermo Fisher). The gene fragments were designed such that the CMAH and GGTA1 genes would be expressed in a bi-cistronic vector configuration, with or without fluorescent tags added to either the N-terminus or the C-terminus of the genes for convenient expression monitoring. Tags were also needed for immunofluorescence, as there were no commercially available CMAH or GGTA1 specific antibodies. Green fluorescent protein (GFP) with a 5′IRES (internal ribosome entry site) was incorporated into all constructs to select GFP-positive cells expressing the genes of interest and monitor their cellular localization. Lyophilized DNA fragments in an intermediate cloning vector were reconstituted in 8 µL of water and digested with SalI and NotI restriction endonucleases to release the desired fragment, then separated on an agarose gel (1%). Each fragment was ligated into the vector between SalI and NotI sites. The ligation reaction was used to transform chemically competent *E. coli* TOP10 cells. Two to four individual colonies were picked for Qiagen miniprep. After positive sequence verification of the miniprep, one clone was selected for large-scale plasmid preparation (Qiagen maxiprep). The whole plasmid was sequenced and confirmed to have the correct orientation. Additionally, a control vector with GFP expression cassette-only was generated.

### 2.3 Engineering of mAb expressing CHO cell line to express GGTA1 and CMAH

Using electroporation, individual vector constructs were transfected into the CL1 cell line. The cells were cultured in a selective medium containing puromycin and methotrexate (MTX) 3 days post-transfection. Cells were cultured in either 24-well or 96-well plates (Corning, NY), 125 mL shake flasks (Corning, NY), or 50 mL TubeSpin^®^ tubes (TPP, Trasadingen, Switzerland) at 37°C, 5% CO_2_, and 85% humidity. Cells were passaged twice a week at 4 × 10^6^–5 × 10^6^ cells/mL concentrations. Once pools achieved >90% viability and maintained consistent doubling times, they were evaluated in fed-batch cultures to evaluate critical quality attributes.

### 2.4 Single-cell cloning

GFP-expressing CHO cells were sorted on a BD FACSAria™ cell sorter (Becton Dickinson, Franklin Lakes, NJ) into 96-well plates with one cell per well to identify single-cell clones. Plates were imaged on a high-throughput CloneSelect™ Imager (Molecular Devices, Sunnyvale, CA) to verify clonality on Day 0 and monitor clone expansion on Days 7 and 14 post-sort before scaling up to 50 mL spin tubes. Once the clones achieved >90% viability and maintained consistent doubling times, they were evaluated in fed-batch cultures.

### 2.5 Fed-batch cultures

Fed-batch cultivation was performed in 50 mL TubeSpin^®^ tubes (TPP, Trasadingen, Switzerland) to determine the critical quality attributes (productivity, glycans and gene expression) exhibited by the clones. Briefly, individual tubes were set up with a working volume of 15 mL of production medium with glucose as the sugar source, incubated at 36°C, 5% CO_2_, 85% relative humidity, and shaken at 225 rpm with a 50 mm orbital diameter in an ISF4-X incubator (Kuhner AG, Basel, Switzerland). Cultures were inoculated at 10^6^ cells/mL and fed a bolus on Days 3, 6, and 8. Glucose concentration was maintained between 10 and 12 g/L through supplemental feeding using a 50 g/L stock solution. Additionally, on day 3, 1 mM uridine, 2 µM manganese(II) chloride (MnCl2), and 5 mM galactose (UMG cocktail) were added to the bolus feed (Gramer et al., 2011). During cultivation, samples were taken on Days 0, 3, 6, 8 and 10 to analyze cell and mAb concentrations, mRNA expression, RNA sequence, and glycans. Cell concentrations and viability were measured using a Vi-Cell XR counter (Beckman Coulter, Brea, CA). Antibody concentrations were determined by protein A affinity high-performance liquid chromatography (Protein A HPLC; Waters™, Milford, MA, United States). Cell culture supernatants were purified *via* affinity chromatography (Atoll GmbH, Weingarten, Germany). Size Exclusion Ultra High-Performance Liquid Chromatography (SE-UHPLC) was performed using Waters™ Acquity H-Class UPLC^®^ with an Acquity BEH 200 column, 200Å, 1.7 μm, 4.6 mm × 150 mm (Waters™, #186005225) monitoring at 220 nm with a tubular ultraviolet (TUV) lamp.

### 2.6 Glycan and molecular attribute analysis

Liquid chromatography with tandem mass spectrometry (LC-MS/MS) analysis was performed for glycan and molecular attribute analysis following titer determination and protein purification. The pepsin digest of purified protein was optimized using appropriate enzyme ratio and digestion times to release one prominent glycopeptide. The digest was prepared either manually or by an automated method using Agilent’s UHPLC (1290 Infinity) autosampler. The separation of glycopeptides derived from the Fab and Fc were optimized to determine the site-specific glycan distribution with a short HPLC gradient for peptide mapping. A high-resolution mass spectrometer (Thermo Scientific LTQ-Orbitrap™ or Themo Scientific QE) was used to generate MS/MS fragments of glycopeptides. The glycopeptides at each site were identified and quantified from the LC-MS/MS data using the MassAnalyzer program (Shah et al., 2014). For molecular attribute analysis, the sequence of the RB is known. The theoretical masses of unmodified and post-translationally modified peptides were calculated using bioinformatics tools (Zhang et al., 2020). Site specification modifications were confirmed based on comparing MS/MS fragmentation data with predicted fragmentation data (Zhang and Shah, 2010).

### 2.7 Expression analysis of CMAH and GGTA1

For mRNA expression analysis, a QuantiGene™ RNA MultiPlex (Thermo Fisher Scientific) gene expression assay was used (Kang et al., 2015). Briefly, cell pellets from 5 × 10^5^ viable cells were lysed using 1X lysis buffer, supplemented with proteinase K (stock concentration 50 mg/mL) and incubated at 50°C for 1 h. Cell lysates were stored at −80°C. A gene-specific probe set and a capture bead set (Panel 41211-CMAH, GGTA1, and control genes GUSB and TBP) were custom synthesized (Affymetrix, ThermoFisher). According to the manufacturer’s protocol, frozen lysates were thawed and processed for mRNA quantification in 96 well plates. The plates were read using the FLEXMAP 3D^®^ (ThermoFisher Scientific) instrument and xPONENT^®^ 4.2 software. Signals recorded as the mean fluorescence intensity (MFI) generated from each bead are proportional to the amount of each mRNA captured on the surface of each generated specific probe set. Next, gene expression was normalized using the expression of the constitutive genes TATA-binding protein (TBP) and glucuronidase beta (GUSB). Using normalized MFI data, the fold expression in engineered clones was compared to control clones generated by transfecting the CL1 cell line that contained the GFP vector alone.

### 2.8 Immunofluorescence and confocal microscopy

To determine if the tagged exogenous enzymes, GGTA1 and CMAH, expressed as expected, immunofluorescence staining and confocal microscopy were performed. Primary antibodies were: anti-GFP (ab13970; Abcam; 1:1000), anti-FLAG (14793, Cell Signaling Technology; 1:800), anti-V5 (R960-25, ThermoFisher; 1:500), and as a Golgi marker anti-GPP130 (610823, BD Biosciences; 3:1000). Corresponding secondary antibodies were: Alexa Fluor 488F(ab′)2-donkey anti-chicken IgY (703-546-155, Jackson ImmunoResearch; 1:500), Alexa Fluor 568F(ab′)2-goat anti-rabbit IgG (A-21069, ThermoFisher; 1:500), and APC F(ab′)2-goat anti-mouse IgG (A10539, ThermoFisher; 1:250).

Cells were first rinsed with phosphate buffered saline (PBS, pH 7.4) and fixed in 4% paraformaldehyde (diluting 8% PFA, 157-8-100; Electron Microscopy Sciences) for 15 min. Rinsing twice with PBS, cells were then permeabilized using 0.05% Triton X-100 (93418; Sigma-Aldrich) in PBS for 15 min. After again rinsing twice with PBS, a blocking step was performed using equal parts mouse Fc and human Fc (at 1:100) in 1% goat serum in PBS for 30 min. Incubation with primary antibodies was then carried out in 1% goat serum in PBS for 2 h. After rinsing twice with PBS, cells were incubated with secondary antibodies and the DNA stain Hoechst (1:1000) in 1% goat serum in PBS for 30 min. Cells were rinsed twice with PBS and stored in PBS until ready for imaging. Images were captured on an Opera Phenix High Content Screening system (PerkinElmer) using a 63x objective with z-stack acquisition.

### 2.9 Stability assessment

To evaluate the stability of critical quality attributes (transgene, expression, and product quality) as a function of *in vitro* cell age a stability assessment was performed. For a stability study, a subset of 20 randomly chosen clones with high productivity (>1 g/L) and varying glycan profiles was selected. A single frozen cell bank vial for each of the 20 clones at predetermined population doubling levels (PDLs) were thawed and cultured in a growth medium containing MTX and puromycin. Cells that were frozen at 15, 30, 50, and 65 PDLs were thawed and propagated in the selective medium for an additional 25 PDLs to emulate the large-scale seed train. Subsequently, these clones were tested in 10 mL fed-batch cultures in 50 mL spin tubes using a non-selective medium (no MTX and puromycin) in spin tubes. The coefficient of variation (%CV) for each attribute (titer, mRNA of exogenous genes and glycan profile, across PDLs) was calculated based on the formula below:

$$\%CV=\frac{\sigma }{µ}$$

where *σ* is the standard deviation of a given attribute across 15, 30, 50, and 65 PDLs and *µ* is the mean of a given attribute across 15, 30, 50, and 65 PDLs.

### 2.10 Bioreactor cultures

All benchtop studies were performed in 3 L stirred tank bioreactors (Applikon, Foster City, CA) with an initial 1.5 L working volume in fed-batch mode. Cultures were fed a bolus on Days 3, 6, and 8. Glucose concentration was maintained between 10 and 12 g/L through supplemental feeding using a 50 g/L stock solution. Additionally, on Day 3, UMG was added to the bolus feed. Bioreactor parameters, dissolved oxygen (DO) concentration, pH, and temperature, were monitored and controlled using an Applikon in-Control (Applikon, Delft, Netherlands) controller. The pH was controlled through CO_2_ or 1 M Na_2_CO_3_ addition. Dissolved oxygen was maintained by sparging oxygen through a drilled pipe and a sintered sparger. Viable cell density and viability were measured using a Cedex HiRes Analyzer (Roche CustomBiotech, Penzberg, Germany). Metabolites, glucose, lactate, glutamate, glutamine, Na+, K+, Ca2+, and ammonia were analyzed using the Cedex Bio HT Analyzer (Roche CustomBiotech, Penzberg, Germany). Osmolality was measured based on freezing point depression using the Advanced Instruments OsmoPRO (Advanced Instruments, Norwood, MA). External pH, pCO_2_ and pO_2_ were analyzed using a Siemens RAPIDLab 1260 (Siemens, Forchheim, Germany). mAb titers were measured using an HPLC Protein A method, and mRNA was profiled using the QuantiGene^®^ based assay described above.

### 2.11 Functional bioassays

The potency of the mAb produced by selected clones- A-29 and A-52 from the small-scale spin tube and benchtop bioreactor were assessed in antibody-dependent cellular cytotoxicity (ADCC) assays and for binding to FcγRIIIa (158V) and cell surface antigen. ADCC was evaluated using the Eurofins DiscoverX Killing Immune-Lysis Reaction (KILR) assay method (Saleem et al., 2020). Briefly, the assay used the commercially available target cells, U2OS KLR, (Eurofins DiscoverX, Fremont, CA) that overexpress the cell surface target and a KILR reporter protein, a constitutively expressed protein tagged with enhanced ProLabel (ePL), and a β-galactosidase (β-gal) enzyme fragment. The target cells were opsonized with a concentration range of purified mAb and RB. The opsonized target cells were incubated with the natural killer effector cell lines, NK92-M1, stably transduced to express human CD16. The tagged KILR protein released from lysed target cells in the media was detected by detection reagents containing the enzyme acceptor fragment of the β-gal reporter. This led to the formation of the active β-gal enzyme that hydrolyzed the substrate to give a chemiluminescent output, detected on a luminometer. Dose-response curves were fit to a four-parameter logistical regression line using SoftMax^®^ Pro Software (Molecular Devices, Sunnyvale, CA), and relative binding was determined by comparing the half-maximal effective concentration (EC50) of a sample to that of a reference standard. All samples were tested in triplicate for each assay, with the mean values reported as relative ADCC activity.

FcγRIIIa (158V) activity was determined using AlphaLISA assays (Perkin Elmer). FcgRIIIa (158V) GST fusion protein and biotinylated human IgG1 was generated at Amgen, Inc. The assay was performed as described previously (Seo et al., 2018). The sample binding relative to the RB was determined using four-parameter logistical regression with SoftMax^®^ Pro Software (Molecular Devices). Results were reported as percent relative binding values.

Relative cell surface target binding was determined using flow cytometry. Briefly, CHO cells overexpressing the cell surface target were incubated with increasing concentrations of purified mAb from the two selected clones and RB at 4°C for 1 h. After washing with wash buffer (phosphate buffer saline (PBS) with 2% fetal bovine serum (FBS), cells were incubated with secondary antibody, allophycocyanin (APC) AffiniPure Goat Anti-Human IgG, Fcγ fragment specific (Jackson Immuno Research Laboratories) for 30 min at 4°C. Cells were then washed and resuspended in a wash buffer containing the dead cell stain Sytox™ Blue (Thermo Fisher). Samples were analyzed on a Becton Dickinson LSR Fortessa™ Cell Analyzer and the APC MFI was analyzed using FlowJo™ software (FlowJo, LLC). Dose-response curves were fit to a four-parameter logistical regression line using SoftMax^®^ Pro Software (Molecular Devices, Sunnyvale, CA). Relative binding was determined by comparing the half-maximal inhibitory concentration (IC50) of a sample to that of a reference standard. All samples were tested in triplicate for each assay, with the mean values reported as relative target binding.

## 3 Results

### 3.1 Characterization of the RB glycan profile

The type and distribution of glycoforms in the RB were analyzed for different lots of RB (Table 1). Glycans located at the two glycosylation sites on the Fab and Fc were a complex mixture of glycoforms. Besides the terminal glycoforms commonly found on antibodies derived from CHO-based processes, namely high mannose (HM), terminal sialic acid, N-Acetylneuraminic acid (NANA), galactose, and fucosylated species, the RB also contained Fab and Fc terminal glycans characteristic of murine cell products, particularly Galα-1,3-Gal (α-Gal) and N-glycolylneuraminic acid (NGNA). Interestingly, the Fc glycans were mainly complex N-glycans terminating with GlcNAc and galactose residues, while the Fab glycans were predominantly α-glycosylated structures. Nearly all glycans contained the core α1,6-fucose.

**TABLE 1:** Glycan distribution from US and EU lots of the reference biologic (RB) that has Fc and Fab glycosylation sites. The subscripts indicate the EU or US RBs where the results were different. α-Gal and Sialylated (NGNA) are not made in CHO cell lines. N-Acetylneuraminic acid (NANA), N-glycolylneuraminic acid (NGNA).

Glycan distribution %	Fab	Fc
aFuc (Complex + Hybrid)	0.6–1.1	1.7–2.1
HM (M5-M8)	0.8–1.4	7_US_ - 12_EU_
Terminal Galactose (all)	85–89	43_EU_ - 48_US_
α-Gal	76–89	2.6–3.2
Sialylated (NANA)	1.0_EU_–3.9_US_	0
Sialylated (NGNA)	29_EU_–39_US_	0.3–0.4

### 3.2 Genetic engineering of CHO cells with murine GGTA1 and CMAH genes

CHO cells were engineered to express relevant glycosylation enzymes that would produce proteins with glycan profiles similar to that of the murine-derived RB. As overexpression of ST6GAL1 can lead to undesirable elevated sialylation on both the Fc and Fab sites (Onitsuka et al., 2012; Lin et al., 2015; Yin et al., 2015), we only introduced CMAH and GGTA1. GGTA1 transfers galactose from uridine diphosphate galactose (UDP-gal) to the terminal galactose residue of N-acetylgalactosamine, producing α-Gal, the dominant glycan form in the RB. There was also a possibility that ST6GAL1 would compete with GGTA1 for the same terminal β-1,4-linked galactose to transfer sialic acid from CMP-sialic acid. Finally, it was considered that adding a third glycosylation-associated gene could adversely impact cell line stability and productivity due to metabolic overload.

**FIGURE 2:**
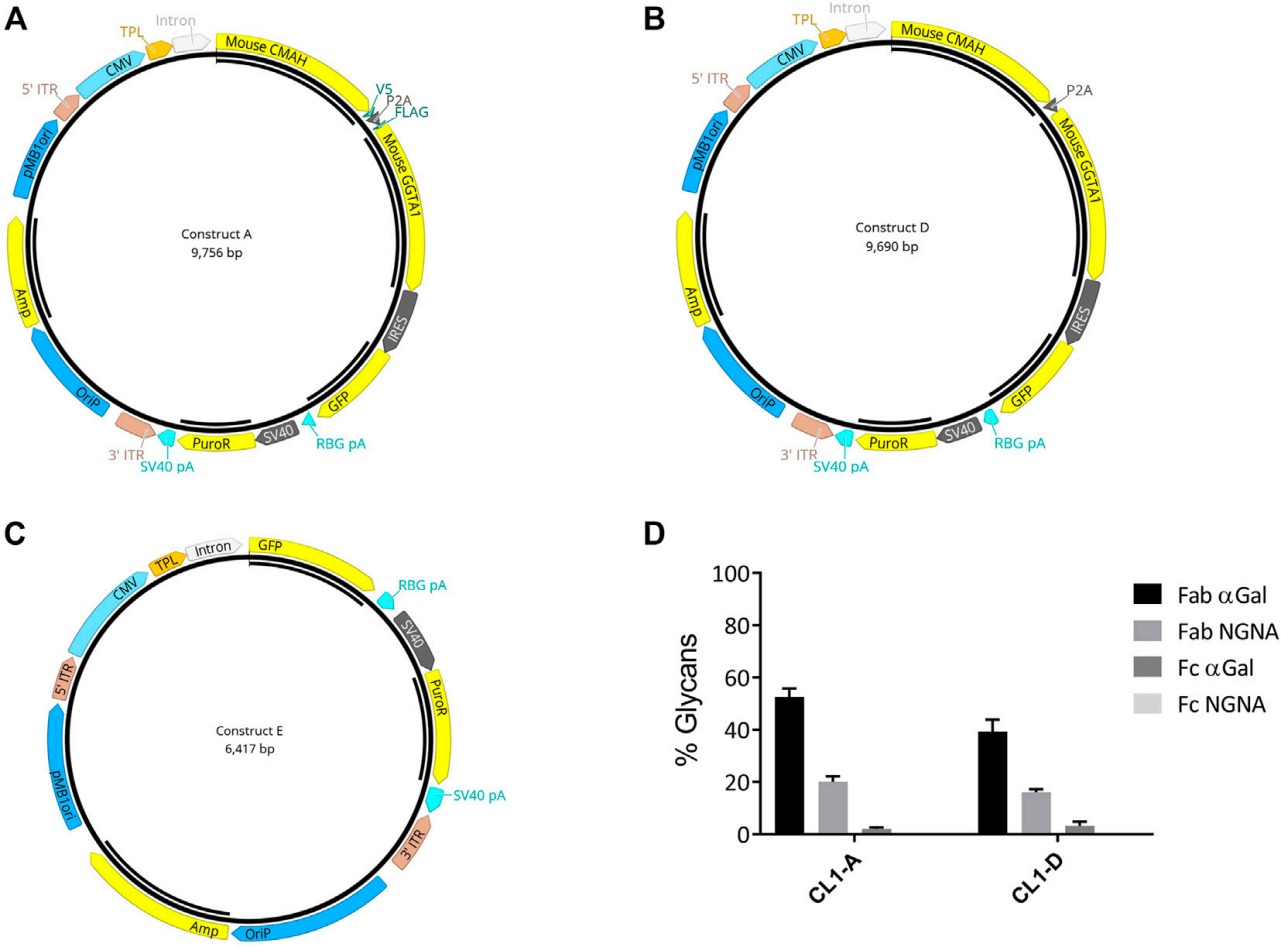
Strategy for biosimilar cell line development. Both constructs A and D contain mouse CMAH and GGTA-1. The two genes were joined by the furin 2A self-cleaving peptide. **(A)** In Construct A, CMAH was fused with a C-terminal V5 tag and GGTA1 was fused with an N-terminal FLAG tag. Green fluorescent protein (GFP) with a 5′ IRES (internal ribosome entry site) was incorporated in Constructs A and D. **(B)** Construct D was similar to Construct A without Flag and V5 tags. **(C)** Construct E was engineered to express GFP only as control. **(D)** LC- MS/MS analysis was performed to determine the glycan profiles (%glycans) from day 10 fed-batch harvest samples from the cell pools generated by transfection of constructs A and D (CL1-A and CL-D) respectively. Selective glycans-Fab αGal, Fab NGNA, Fc αGal, and Fc NGNA are shown. percent change of % relative glycans Fab (α-Gal and NGNA) and Fc (α-Gal and NGNA) when compared to CL1-E control pools. The error bars of the percent changes were derived from the relative errors of control and pools generated from different transfections of individual vectors (*N* = 2).

Native GGTA1 is a type II Golgi membrane-bound protein with a short amino-terminal cytoplasmic tail, a single transmembrane domain, and a large COOH-terminal domain in the Golgi lumen (Boix et al., 2002); whereas CMAH is present in the cytoplasm. Glycosylation follows a sequential, step-wise pathway. Therefore, the relative localization of glycosylation-related enzymes significantly impacts the types of glycan structures produced by the cell. Thus, a vector was designed to express GGTA1 and CMAH with N- and C-terminus tags (Flag and V5) to allow monitoring of proper subcellular localization. (Construct A; Figure 2A). As the tag sequences could potentially interfere with the proper folding or activity of the enzymes, a vector containing CMAH and GGTA1 without tags was also designed (Construct D; Figure 2B). In both vectors, CMAH and GGTA1 were joined by the self-cleaving peptide furin-2A, to maintain the 1:1 stoichiometry of expression of the two genes. Vectors also included GFP flanked with an N-terminal IRES downstream of the gene of interest. The presence of IRES allowed the expression of both upstream genes and GFP from a single promoter. Therefore, visualization of GFP in a cell would signify the presence of both CMAH and GGTA1 in 1:1 stoichiometry. Additionally, a control vector that contained GFP alone was designed as control (Construct E; Figure 2C).

The CHO cell line CL1 was then transfected with the three constructs A, D and E, generating pools CL1-A, CL1-D, and the control, CL1-E. The stably transfected pools were evaluated in a fed-batch production assay for various critical quality attributes, e.g., productivity, glycan, and expression of engineered genes. Optimal culture performance, including end-of-production titer, viable cell density (VCD), and cell viability, was observed from all pools (data not shown). LC-MS/MS analysis of purified mAbs confirmed an up to 50% and 20% increase in the percentage of Fab α-Gal and NGNA glycans, respectively (Figure 2D).

### 3.3 Overexpression of GGTA1 and CMAH increases both Fab and Fc alpha-gal and NGNA in engineered CHO cell lines

**FIGURE 3:**
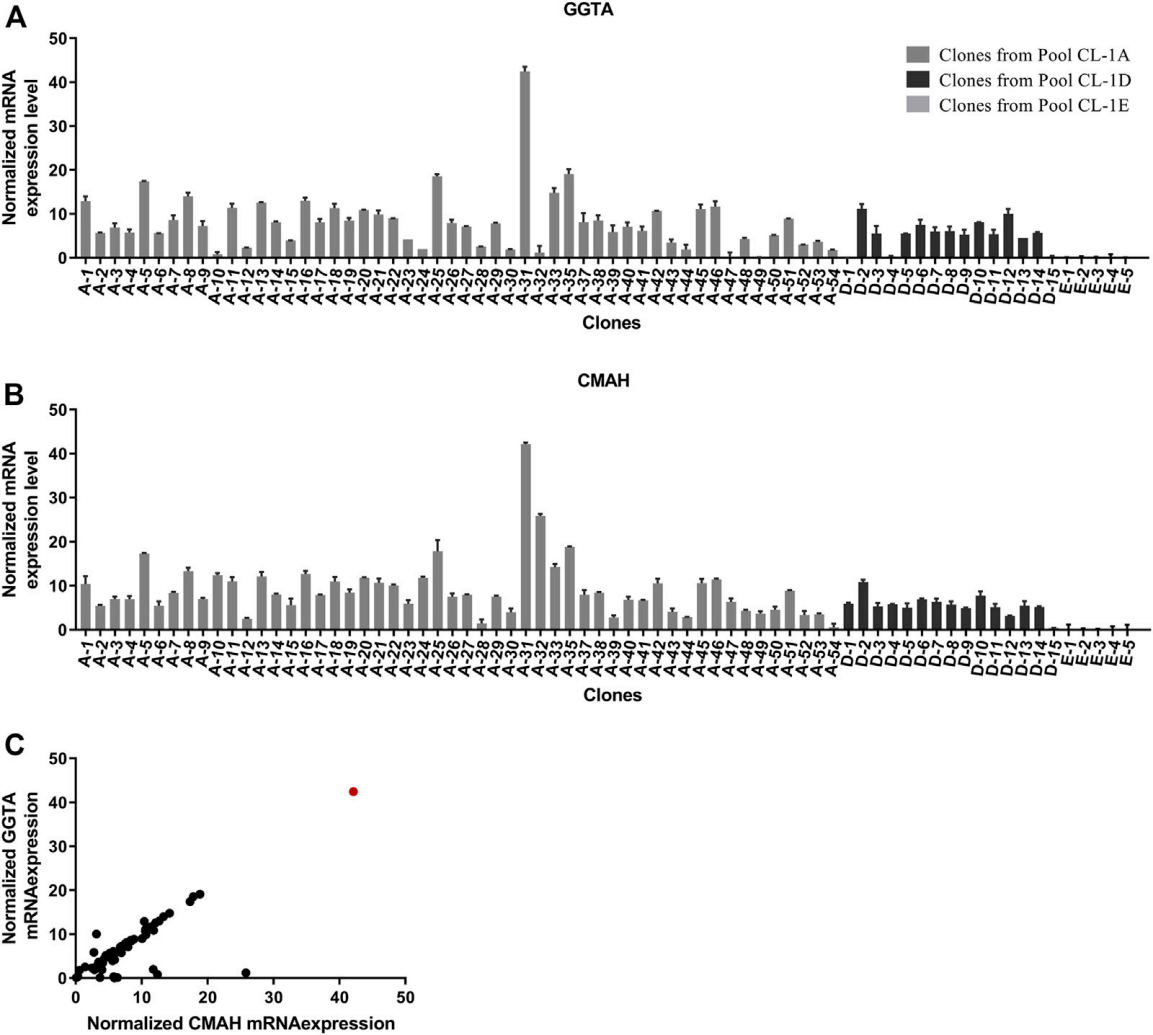
Profiling of GGTA and CMAH expression and functionality in cell clones. **(A, B)** Day 8 mRNA transcript expression of overexpressed genes in CHO CL1 derived clones. The mRNA expression was normalized to constitutive genes Gusb and Tbp. Overexpression of **(A)** GGTA1 and **(B)** CMAH was determined in fed-batch cultures across multiple clones generated from Constructs A and D compared to Control E **(C)** Bi-cistronic vector configuration resulted in linked expression of both GGTA1 and CMAH. Day 8 mRNA transcript expression of overexpressed genes in CHO CL1 derived clones. The mRNA expression was normalized to constitutive genes Gusb and Tbp. Clone 31 is shown in the red circle.

Single cell clones were identified by FACS sorting on GFP positivity and scaled up to 20 mL scale. A total of 72 clones with an optimal and consistent doubling time (<30 h), even in the presence of double selection (MTX and puromycin), were selected for further evaluation. The 72 single clones comprised 67 experimental clones (CL1-A and CL1-D) and five control clones (CL1-E). These clones were evaluated in a 10-day fed-batch culture for growth, productivity, and glycan profile. To increase both Fab and Fc galactosylation, a bolus feed of UMG substrates was added on Day 3. During the production run, time-course mRNA profiling (Days 3, 6, 8, and 10) revealed a consistent mRNA pattern of the exogenous genes CMAH and GGTA1 throughout the process. As expected, control clones did not express CMAH and GGTA1, whereas experimental clones expressed 2 to 43-fold higher normalized mRNA levels of CMAH and GGTA1 normalized to the constitutive genes GUSB and TBP (Figures 3A, B). Using a self-cleaving furin-2A, a bicistronic configuration of the two genes successfully demonstrated the precisely controlled and 1:1 stoichiometry of CMAH and GGTA1 expression in a majority of the clones (Figure 3C). Comparable growth patterns between control and experimental clones were observed, demonstrating that expression of exogenous CMAH and GGTA1 was not detrimental to cell viability or growth (data not shown). Only clone 31 showed a poor growth profile throughout the fed-batch experiment. Clone 31 was able to reach a maximum viable cell density of 5 × 10^6^ cell/mL during the fed-batch. In contrast, all other clones ranged from 10–26 × 10^6^ cell/mL. This observation could be explained by the exceptionally high mRNA levels of GGTA1 and CMAH, potentially leading to increased metabolic load and, in turn, poor growth performance (Figure 3C).

**FIGURE 4:**
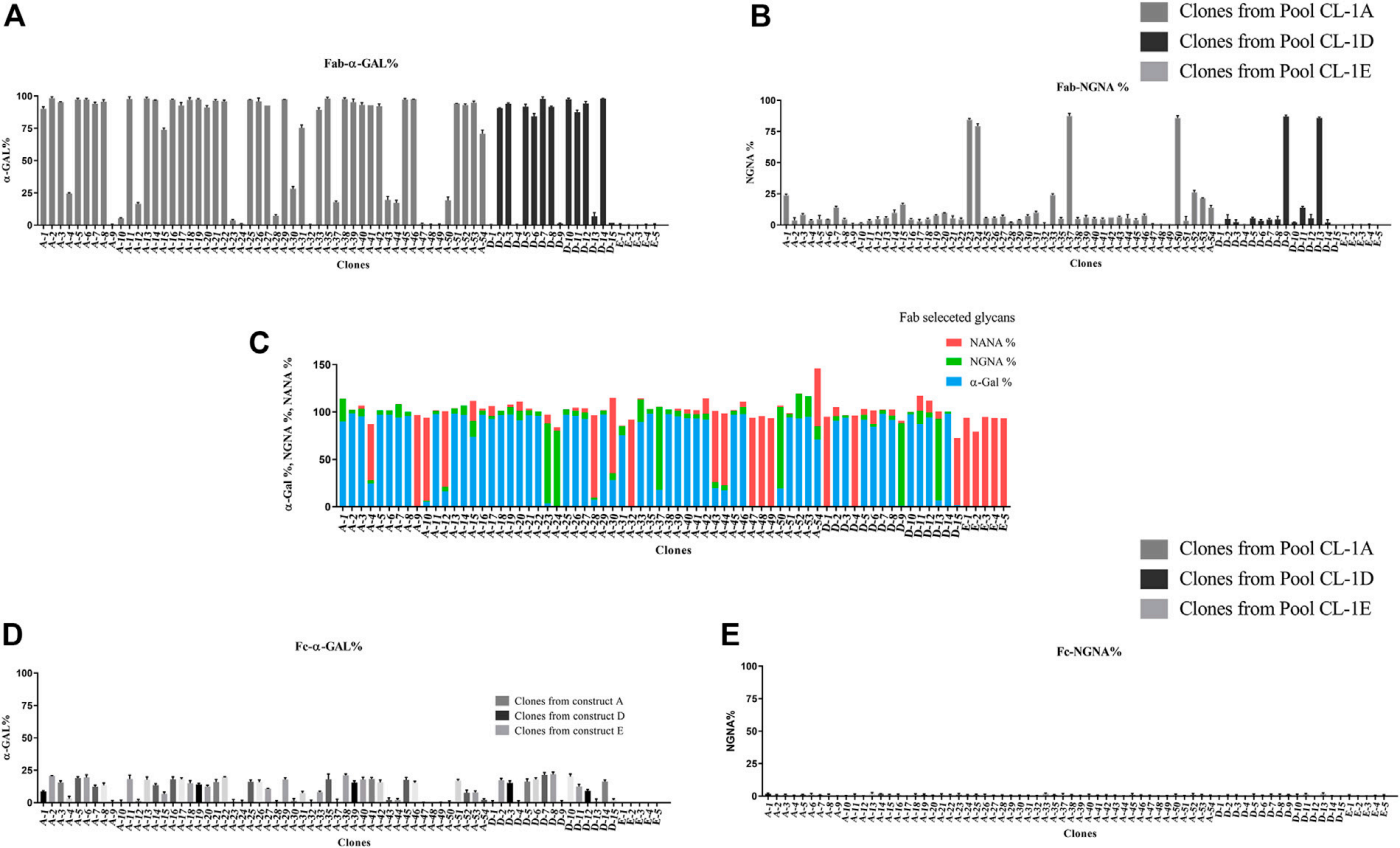
LC-MS/MS analysis was performed for glycan analysis on day 10 of fed-batch harvest samples. **(A)** Fab-α-Gal and **(B)** Fab-NGNA was observed in clones overexpressing the two genes of interest compared to the controls. **(C)** Inverse relation between Fab % α-Gal-NGNA-NANA was observed. **(D)** Compared to the control (E clones), an increased level of Fc-α-Gal was observed in various clones overexpressing the two genes of interest. **(E)** There was a negligible presence of Fc-NGNA for all the clones. Each data represents culture samples run in duplicate; error bars represent standard deviations.

**FIGURE 5:**
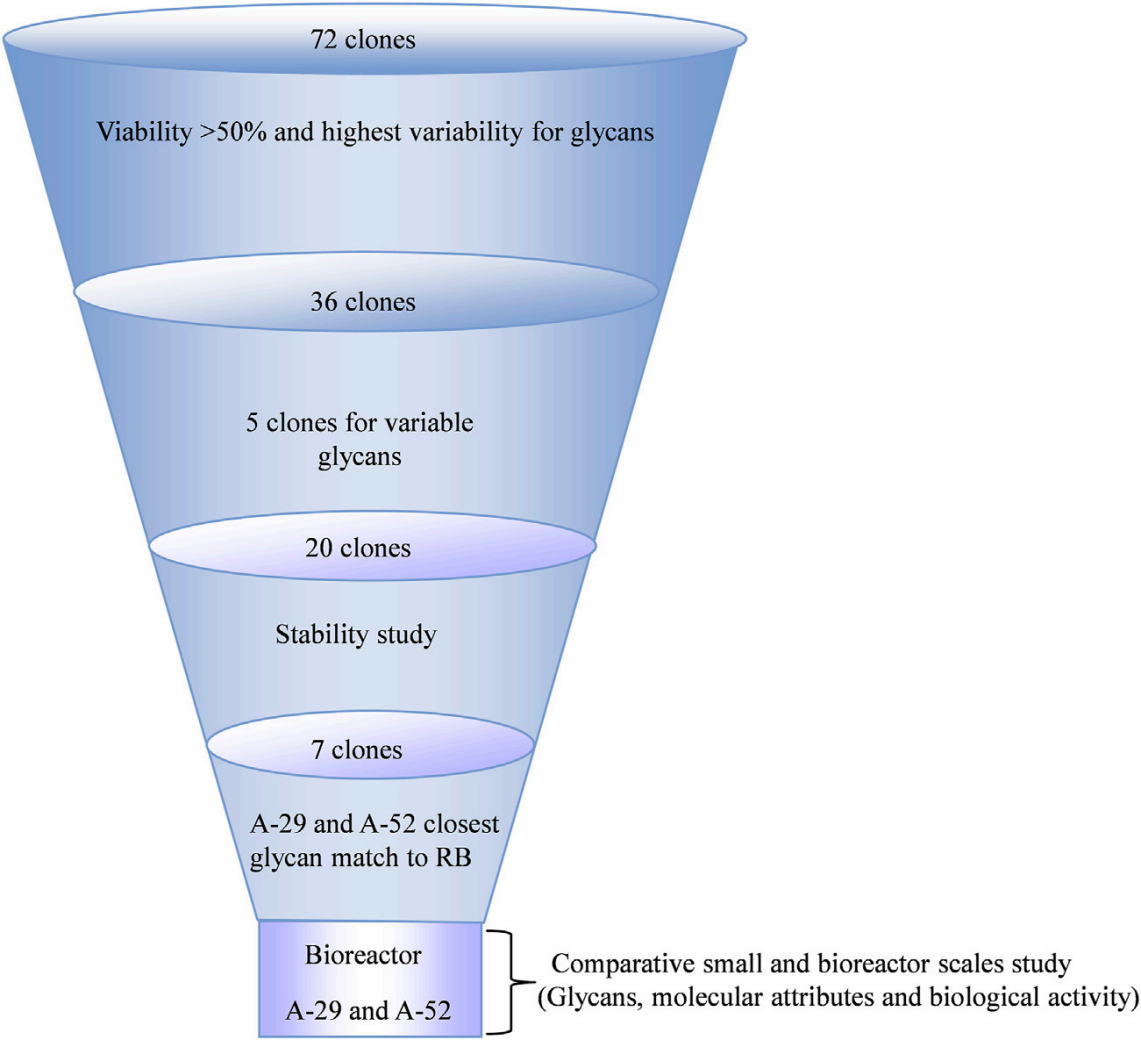
Selection and workflow of the clone selection.

Next, the glycan profile on Day 10 harvested mAb was assessed. Despite the 1:1 stoichiometry of CMAH and GGTA1 mRNA expression, the majority of clones had high α-Gal and lower NGNA, which was consistent with the RB profile. Specifically, most experimental clones had high levels of Fab α-Gal (ranging from 0.9% to 98%), with overall lower levels of Fab NGNA (ranging from 0.2% to 87%), similar to the RB (Figures 4A, B). Upon collectively looking at the Fab α-Gal, NGNA and NANA, an inverse relationship was observed (Figure 4C). In contrast Fab glycans, Fc α-Gal ranged from 0% to 25%, with a negligible expression of Fc NGNA (Figures 4D, E). The high level of Fabs α-Gal and NGNA dramatically increased the possibility of a clone producing an antibody within the RB glycan profile range. The selection of clones and workflow is shown in Figure 5.

### 3.4 Localization of exogenous GGTA1 and CMAH in engineered CHO cells

**FIGURE 6:**
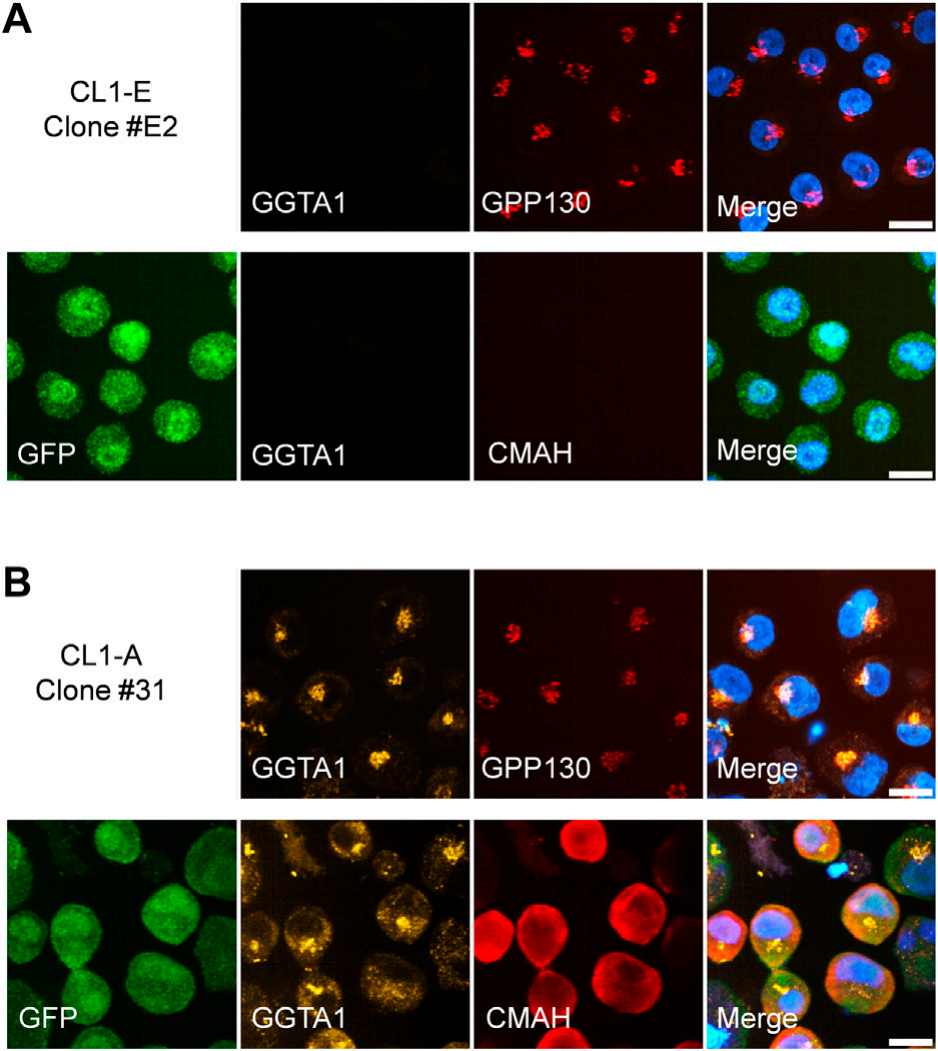
Localization of exogenous GGTA1 and CMAH in stably transfected clones. **(A)** CL1-E clone #E2 shows only the presence of GFP. Top row: staining with mouse monoclonal anti-FLAG M2 and rabbit polyclonal anti-GPP130 detects GPP130 but not FLAG-GGTA1. Bottom row: additional staining with mouse anti-GFP and rabbit monoclonal anti-V5 (instead of anti-GPP130) detects GFP but not CMAH-V5. **(B)** Expression of FLAG-GGTA1 and CMAH-V5 is detected in CL1-A clone #31. Top row: Colocalization of FLAG-GGTA1 and GPP130 is observed. Bottom row: staining for GFP and V5 show strong cytoplasmic localization for most cells. Hoechst was used to visualize cell nuclei in all cases. All scale bars are 10 μm.

Confocal imaging was used to confirm the proper subcellular localization of endogenously expressed GGTA1 in the Golgi membrane and CMAH in the cytoplasm. Due to the lack of commercially available antibodies against GGTA1 and CMAH, FLAG-GGTA1 and CMAH-V5 fusion protein constructs were utilized (Figure 2A). From CL1-A, four experimental clones were randomly selected, and from construct CL1-E, one control clone was selected. All clones were stained for the cis-Golgi membrane protein GPP130. In the CL1-E control clone, GGTA1 (Figure 6A, top row) and CMAH (Figure 6A, bottom row) were not detected, with only GFP signal visible. In the CL1-A clones, FLAG-GGTA1 was detected in the Golgi region and colocalized with GPP130 (Figure 6B, top row), whereas CMAH-V5 was found localized throughout the cytoplasm (Figure 6B, bottom row). Images from the other three experimental clones are provided in Supplementary Figure S1. These results are consistent with other reports in the literature (Milland et al., 2002; Nystedt et al., 2010), indicating that the localization of the exogenous enzymes GGTA1 and CMAH was similar to that of the endogenous enzymes.

### 3.5 Cell line stability

**FIGURE 7:**
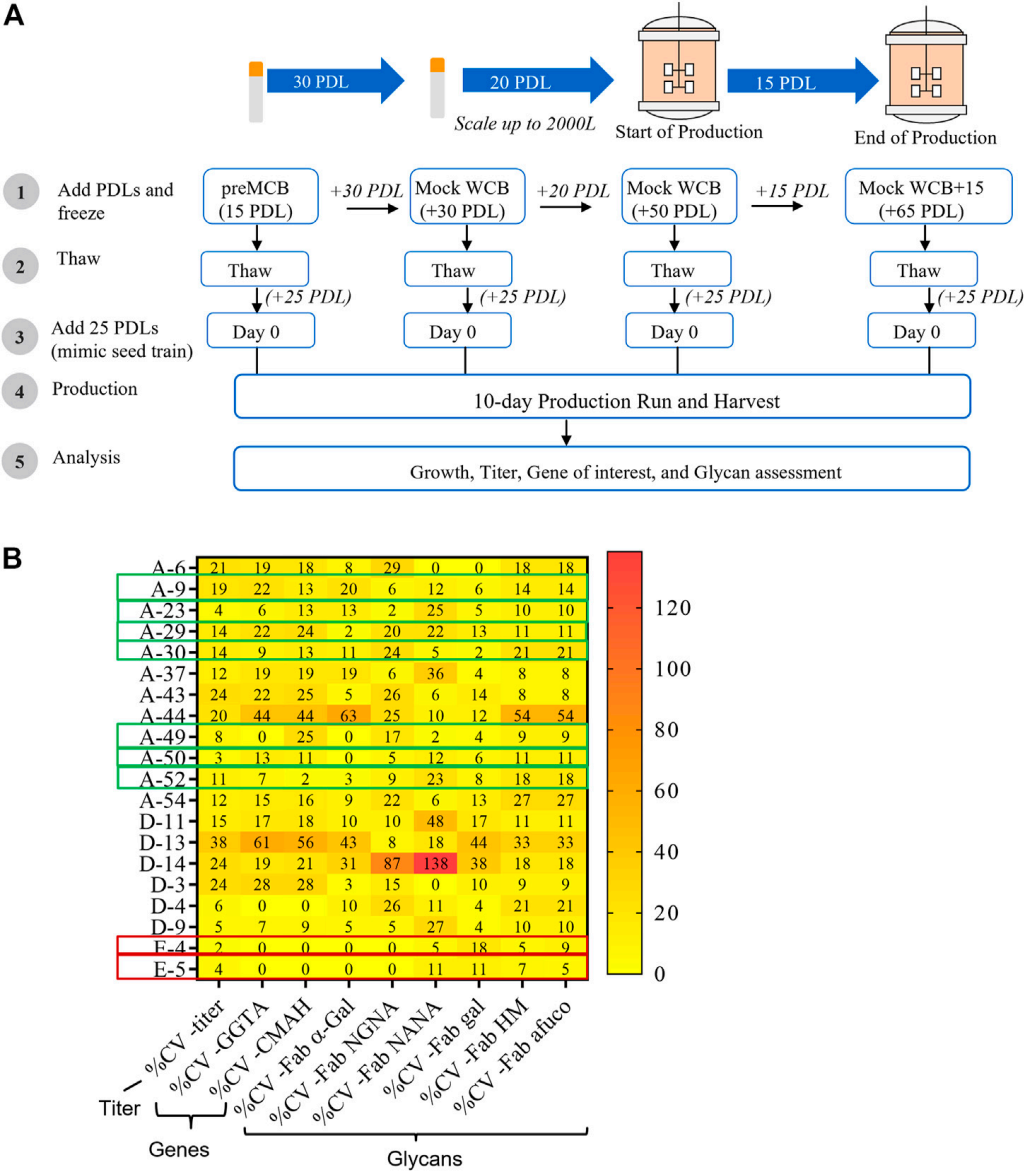
Impact of generational age cell line stability attributes. Cell line stability for 20 clones was assessed by evaluating changes in titer, mRNA expression of the genes of interest, and glycan profile over defined duration and intervals of Population Doubling Levels (PDLs). 20 clones included 18 clones from the CL1-A or CL1-D and two clones from the CL1-E control cell line. **(A)** Schematic of the stability study design. **(B)** The coefficient of variance (%CV) across different PDL for each clone was evaluated for productivity, mRNA expression of genes of interest, and glycans. Seven clones (from either CL1-A or CL1-D) highlighted in the green boxes exhibited acceptable <20% CV for titer and <25% CV for other attributes indicating the stability of various PQAs through age. Similarly, two clones (from CL1-E) highlighted in the red box indicate stability.

A critical criterion for the successful generation of an engineered cell line is the stability of the transgene expression upon extended culture (Bailey et al., 2012). Stability is generally evaluated for a course of 50–60 PDLs, the time required to generate a master cell bank (MCB), a working cell bank (WCB), and execute at a commercially relevant production scale (Figure 7A) (Hammill et al., 2000; Bebbington and Lambert, 1994).

To investigate the genotypic and phenotypic stability of the engineered cell lines, a subset of 20 randomly chosen clones (18 clones from the CL1-A and CL1-D cell lines with high productivity (>1 g/L) and varying glycan profiles and two control clones, CL1-E clones E-4 and E-5) were selected for a stability study using conditions mimicking typical commercial manufacturing conditions (Figure 7A). Cell banks for different clones at 15, 30, 50, and 65 PDLs were thawed and carried for an additional 25 PDLs to mimic the manufacturing scale seed train and then used in small-scale fed-batch experiments in parallel. The coefficient of variance (%CV) was evaluated in various cell performance and critical quality attributes, including growth, titer, mRNA of exogenous genes, and glycan profile, across PDLs. After extensive screening and characterization, seven of 20 clones demonstrated <20% CV for titer and <25% CV for other attributes, including mRNA expression of exogenous genes and the desired glycan profile (Figure 7B). The data suggest that less than half of the total 20 clones were stable, emphasizing the importance of a thorough stability study.

### 3.6 Evaluation of selected clones in bench-scale bioreactors with acceptable productivity and desired product attributes

**FIGURE 8:**
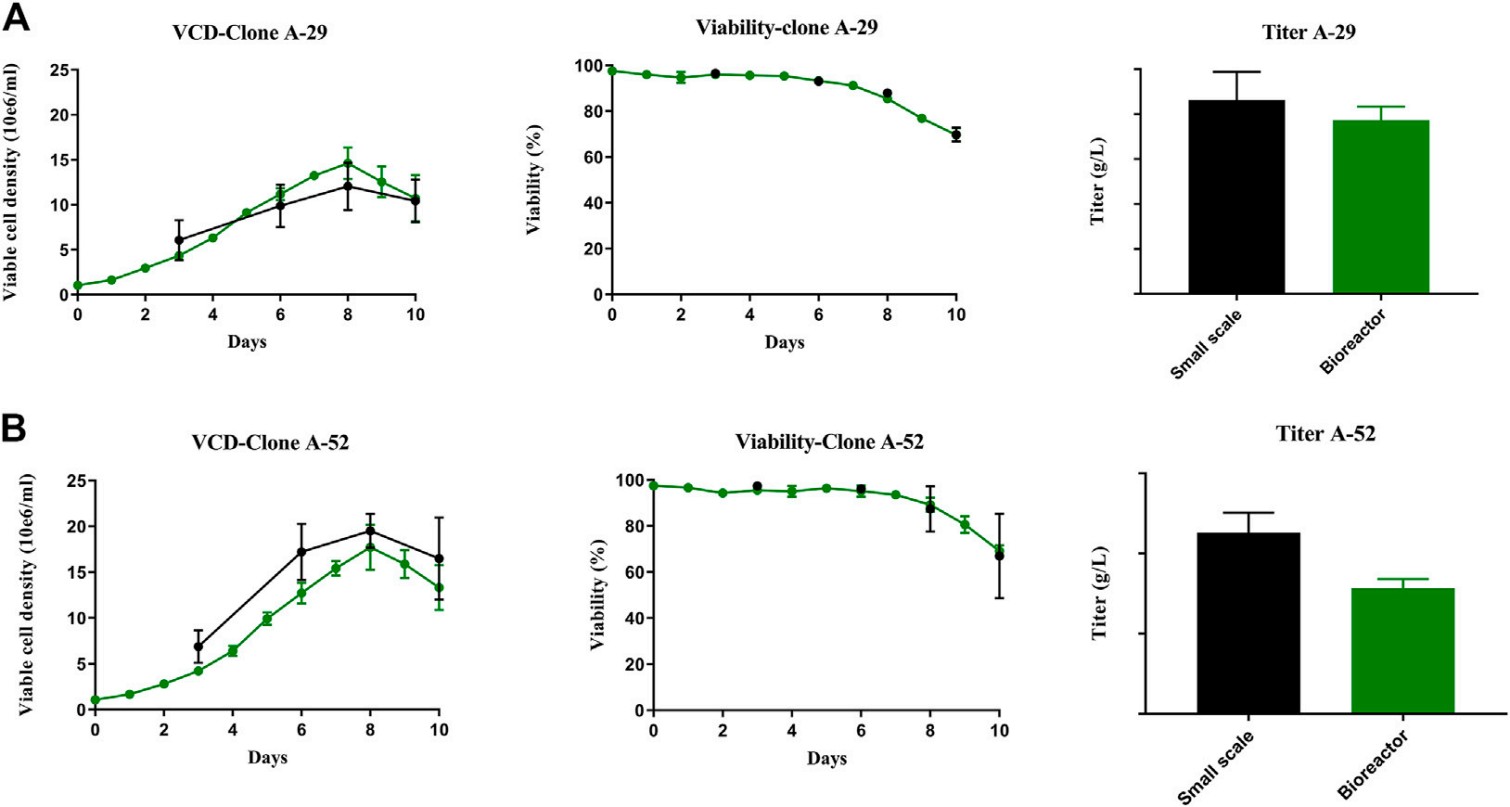
Comparison of process performance across scales. Clone A-29 and A-52 were compared in 10 days fed-batch productions small scale (spin tubes) and benchtop Applikon bioreactors. **(A, B)** VCD, Viability and titer plots for A-29 and A-52 respectively. Error bars represent the standard deviation calculated from data obtained in three independent experiments (*n* = 4) for small scale spin tube and biological replicates (*n* = 3) in bioreactor production.

Of the seven experimental clones that demonstrated maximum stability, clones A-29 and A-52 had the closest glycan profile to that of the RB. Therefore A-29 and A-52 were further characterized to understand their production potential. Clones A-29 and A-52 were scaled up in bench-scale 3 L bioreactors under culture conditions that more closely mimic commercial manufacturing. Similar growth profiles and productivity were observed between small spin tube and bioreactor scales (Figures 8A, B). The viable cell density for both clones reached a maximum of ∼15–18 × 10^6^ cells/mL by Day 8 and slowly declined for the remainder of the culture. The viability ranged from 99% on Day 3 after inoculation of the fed-batch for both the clones to 50% at the end of the culture by Day 10 for both small (spin tube) and bench scales.

Glycan profiling confirmed that clones A-29 and A-52 retained the desired glycan profile under these manufacturing conditions (Table 2). The overall glycan distribution of Fab and Fc glycan forms for both clones A-29 and A-52 was similar to that of the RB. Nonetheless, A-52 most closely resembled the glycan profile of the RB at both the Fab and Fc region. More than 20 glycan species were identified at each Fc (N88) and Fab (N299) glycan site. Four glycan groups were evaluated: galactosylated glycans, sialylated glycans, high mannose glycans, and afucosylated glycans. The levels of α-Gal and β-Gal were calculated as the sum of complex type and hybrid type glycan structures which contain at least one terminal α-Gal and β-Gal, respectively (Table 2). Clones A-29 and A-52 had similar Fab and Fc β-Gal compared to the RB except A-52 in the small-scale run. This difference in glycans at small-scale might be due to the lack of controlled culture conditions at this scale. Clone A-29 had slightly higher α-Gal for both Fab and Fc, whereas A-52 had similar α-Gal levels to the RB. In addition, insignificant sialylation was observed at the Fc region of the RB, and in the case of A-29 and A-52 Fc sialylation (NGNA and NANA) was ≤1.1%. On the other hand, the RB NGNA levels at the Fab region were between 28% and 36%, and clone A-52 matched this range. There was negligible NANA at the Fab region. High mannose was calculated as the sum of M8, M7, M6, and M5. Results show RB had ≤10% HM at Fab whereas Fc HM% was ≤2%, and A-29 and A-52 closely matched these levels of HM%. Afucosylated glycans are comprised of afucosylated complex and afucosylated hybrid types of glycans. A-52 showed afucosylation glycan levels similar to those of the RB, but compared to A-29, A-29 had slightly higher levels of afucosylated glycans. This dataset suggests that overexpression of GGTA1 and CMAH resulted in similar α-Gal and NGNA, respectively to the RB without affecting other glycans such as β-Gal, HM, NANA, and afucose. Together, similar glycan profiles and optimal productivity would qualify these two clones as suitable for biosimilar production.

**TABLE 2:** LC-MS/MS analysis performed on pepsin digest of purified protein on day 10 fed-batch harvest samples for clones A-29 and A-52 for glycan analysis and molecular attributes (relative %). The Fc and Fab glycan profile range of antibodies produced at small and bioreactor scale was compared with reference biologic (RB).

Region	Glycan map (%)		A-29	A-29	A-52	A-52	RB
		Glycan distribution	Small scale (*n* = 4)	Bioreactor (*n* = 3)	Small scale (*n* = 4)	Bioreactor (*n* = 3)	Drug product (*n* = 4)
Fc	Galactosylation	α-Gal	7.37–9.99	6.24–7.51	0.5–2.3	2.5–3.8	0.95–2.31
		β-Gal	45.13–45.57	45.4–48.91	41.8–49.8	50.8–52.6	44.77–52.81
	Sialylation	NGNA	0.65–1	0.5–0.57	0.7–1.1	1.1–1.2	0.09–0.39
		NANA	0–0.02	0.12–0.18	0–0.3	0.3–0.3	0–0.16
	High Mannose	High Mannose	7.42–11.29	8.63–9.83	7.28–7.83	8.58–9.56	9.45–9.65
	Afucosylation	Afucosylation	2.10–2.49	2.05–2.52	1.75–2.17	1.50–1.74	1.09–1.18
		Total Afucosylation	9.57–13.78	10.68–12.35	9.07–9.73	10.17–11.29	10.63–10.75
Fab	Galactosylation	α-Gal	91.41–95.27	90.76–92.53	71.01–74.77	83.84–89.32	77.56–86.60
		β-Gal	6.59–9.02	10.29–11.43	18.04–23.5	9.79–13.86	9.26–22.7
	Sialylation	NGNA	6.15–9.23	10.51–11.16	29.75–37.66	30.91–34.56	28.03–36.21
		NANA	0.52–2.39	0.34–0.4	0.52–11.13	0–0	0–5.36
	High Mannose	High Mannose	1.09–1.40	1.12–1.46	1.01–1.74	1.0–1.22	0.85–1.70
	Afucosylation	Afucosylation	0	0	0	0	0
		Total Afucosylation	1.09–1.40	1.12–1.46	1.01–1.74	1.0–1.22	0.85–1.70

As a part of the analytical similarity assessment, chemical modifications of amino acid residues were evaluated. Any observed modifications can alter antibody functionality and are considered critical molecular attributes. Therefore, all mAb samples and the RB were subjected to digestion and peptide mapping to determine any chemical modifications. As a result, minimal changes in fragmentation and chemical modification were observed in methionine (Met) and tryptophan (Trp) oxidation, glycation of lysine (Lys) amino acid residues in the RB and samples (Table 3). Overall, the minor differences observed were not expected to impact safety and efficacy.

**TABLE 3:** Chemical modifications of amino acid residues were evaluated. Site specific amino acid modifications were confirmed based on the fragmentation of the RB and clones A-52 and A-29. *n* = number of replicates tested.

Attribute	A-29 (*n* = 3)	A-52 (*n* = 3)	RB (drug product) (*n* = 3)
CDR HC Trp52 oxidation	0.18%–0.25%	0.23%–0.30%	0.08%–0.11%
CDR HC Trp94 oxidation	0.25%–0.50%	0.38%–0.41%	0.05%–0.09%
HydroxyLysine (HC Lys123)	0.42%–0.46%	0.41%–0.47%	0.00%–0.01%
Met254 oxidation	0.62%–0.93%	0.58%–0.82%	0.61%–0.80%
HydroxyLysine (HC Lys342)	0.13%–0.22%	0.15%–0.23%	0.00%
Met360 oxidation	0.09%–0.10%	0.10%–0.13%	0.07%
Met430 oxidation	0.80%–0.89%	0.81%–0.98%	0.45%–0.47%

Next, SE-UHPLC was used to quantitate the percentage of high molecular weight (HMW) species in A-29 and A-52. Similar to the RB, A-29 and A-52 were predominately monomers with 1.5% and 1.1% HMW species, respectively, while RB had 0.3% HMW species.

### 3.7 Clones A-29 and A-52 retain similar biological activity to the RB

**FIGURE 9:**
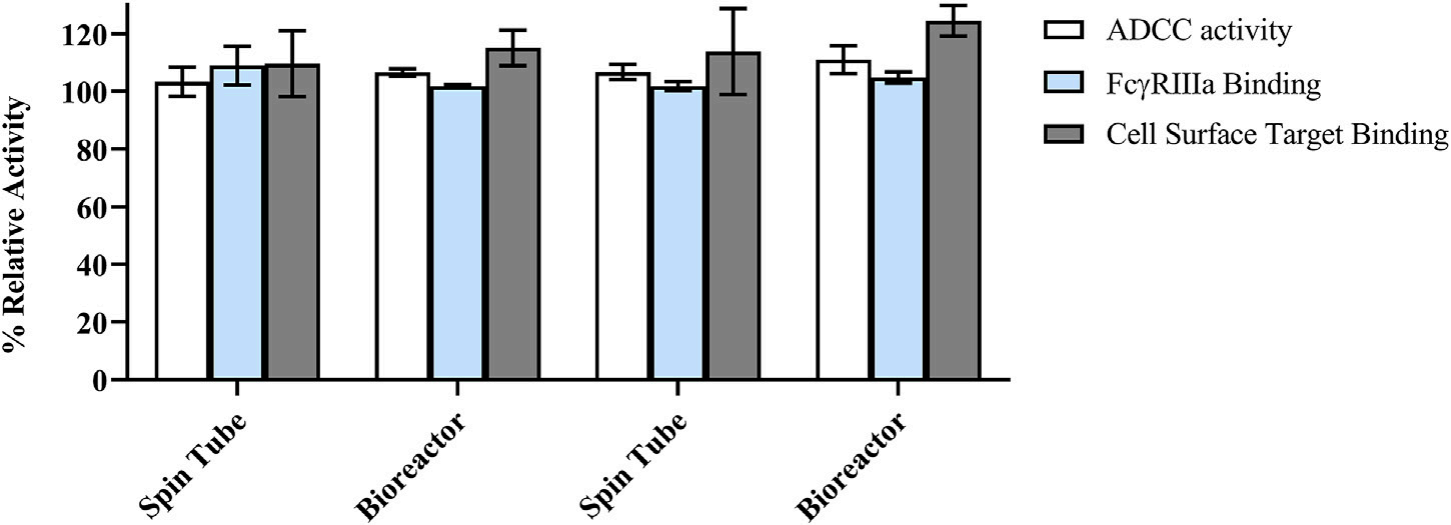
Summary of binding and biological activity of antibodies produced from clones A-29, and A-52 as compared to the reference biologic (RB). ADCC (antibody-dependent cell-mediated cytotoxicity), FcγRIIIa binding activity, and cell surface target binding. Relative activity (%) was calculated against RB reference standard lot. All samples were tested in triplicate for each assay, with the mean values reported as relative activity. The error bars represent the standard deviation calculated from data obtained in independent activity runs (*n* = 3).

The mechanism of action of the RB is mediated by antigen targeting and subsequent ADCC through the interaction of the Fc domain with Fc receptors on the surface of immune effector cells. Hence, FcgγRIIIa (I58V), target antigen binding, and ADCC function of clones A-29 and A-52 were assessed to ensure that biological activity was similar to the RB. Antibodies from both small (spin tube) and bioreactor scales were tested. The antibodies showed similar binding to FcγRIIIa (158V) (blue bars) and cell surface target (grey bars) as the RB (Figure 9). The ADCC activity of the antibodies in the NK92 cells (white bars) was also similar to that of the RB (Figure 9). These data suggest that the antibody retains the expected functionality when expressed in the engineered production cells.

## 4 Discussion

This study demonstrated that cell line engineering can address the challenge of achieving similar glycan profiles between biosimilars produced in CHO cells and a RB produced in murine host cells.

Our initial cloning strategy featured the co-transfection of individual CMAH and GGTA1 vectors in the CL1 cell line. There were two unexpected observations from the use of this strategy. First, the co-transfection resulted in the expression of only one or the other enzyme rather than the desired dual expression of both enzymes. Second, although mRNA expression of either CMAH or GGTA1 was observed in different clones, there was no detectable α-Gal, and NGNA found by LC-MS/MS analysis. This indicated that the enzymes were not translated, were non-functional, or were mislocalized in this system. Interestingly, a recent study also reported similar observations when attempting to overexpress two different glycosyltransferases. It was found that overexpressing two enzymes simultaneously resulted in the truncation and mislocalization of the enzymes (Wang et al., 2020). Our initial results forced us to reconsider our cloning strategy and reinforced the need to demonstrate appropriate subcellular localization of the enzymes. Therefore, in the present study, a single vector was designed with CMAH and GGTA1 joined by furin-2A, a self-cleaving peptide to maintain the 1:1 stoichiometry during the expression of the two genes. In addition, to determine the subcellular localization of CMAH and GGTA1, a construct with tags added to the C- terminus, and N-terminus of the genes, respectively, was designed. With the help of tags in immunofluorescence, the appropriate subcellular localization of the two enzymes was confirmed. This relatively strict compartmentalization of the exogenous enzymes is thought to ensure the efficient biosynthesis of desired glycan structures by providing optimal contact between enzyme, mAb, and sugar nucleotide donor.

Typically, the development of biological products, including both reference biologics and biosimilars, starts with cell line development and the selection and analysis of clones that could potentially result in desired CQAs, followed by media optimization for precise control of the desired CQAs. Instead of adopting this sequential approach, we relied on our understanding of the glycosylation pathway in the development cell line and focused on producing the desired RB glycan profile. Therefore, to ensure adequate levels of galactose needed to make both β-Gal and α-Gal, we added a UMG bolus feed on Day 3 of fed-batch cultivation. The addition of UMG has consistently been shown to increase galactosylation (Gramer et al., 2011; Grainger and James, 2013). In addition, this approach would save us time for later media optimization.

We screened a large number of clones to increase the probability of finding a clone that could have a highly similar glycan profile to that of the RB glycan profile without compromising productivity. Indeed, within our panel of 72 clones, four distinct of glycan profile patterns emerged from small-scale production. Interestingly, the data revealed an inverse relationship between %α-Gal, and %NGNA (Figure 4C). This inverse relationship can have two possible explanations. First, in engineered CHO cell lines, β-1,4Galactosyltransferase 1 (B4GALT1) transfers galactose from UDP-galactose in β-1,4-linkage to the glycan chain on a GlcNAc sugar moiety, then GGTA1 or α-2,3-sialyltransferase 1 (ST3GAL1) sequentially adds α1,3-galactose and/α-2,3- linked sialic acid to β-1,4-linked galactose. Therefore, GGAT1 and ST3GAL1 compete for their common substrate β-1,4-linked galactose (Czlapinski and Bertozzi, 2006). Thus, overexpression of GGTA1 in engineered CHO cell lines can usurp the terminal galactose-containing substrate and convert it to α-Gal. Secondly, it has been shown that glycosyltransferase homomers are assembled in the endoplasmic reticulum. These homomers become disassembled in the Golgi, allowing the formation of specific active glycosyltransferase heteromers. There is no structural information on GGTA1, however, we hypothesize GGTA1 could form heteromers as reported for other glycosyltransferases (Khoder-Agha et al., 2019). Therefore, in the case of excess GGTA1, which may have reduced the association with ST3GAL1, the overall sialylation level in most clones would increase. The diverse panel of 72 clones captured the complexity of the glycosylation pathway and allowed us to identify two clones, particularly clone A-52, with similar glycan profiles to the RB. These clones were found to have similar growth profiles, productivity, analytical similarity assessment, and biological functionality to the RB. Overall, our CHO cell engineering strategy can be applied to the development of future biosimilars to achieve the desired glycan profiles of RBs derived from different host cell species.

## Data Availability

The data analyzed in this study is subject to the following licenses/restrictions: This manuscript utilizes proprietary data. Requests to access these datasets should be directed to the Corresponding Author.

## Abbreviations

ADCC, antibody-dependent cell-mediated cytotoxicity; ADCP, antibody-dependent cell-mediated phagocytosis; B4GALTs, Beta-1,4-galactosyltransferases; CHO, Chinese Hamster ovary; CMAH, cytidine monophospho-N-acetylneuraminic acid hydroxylase; CQAs, critical quality attributes; Fc, fragment crystallizable; FDA, food and drug administration; Fuc, fucose; Gal, galactose; GlcNAc, *N*-acetyl glucosamine; Gusb, glucuronidase Beta; mAb, monoclonal antibodies; NANA, N-acetylneuraminic acid; NGNA, N-glycolyl neuraminic acid; PQAs, product quality attributes; RB, reference biologic; Sia, sialic acid; Tbp, TATA-binding protein; α(2,6) SiaT, α2–6-sialyltransferase.
