# Supplementary figures and images for: Engineering protein glycosylation in CHO cells to be highly similar to murine host cells
Source: Front Bioeng Biotechnol. 2023 Feb 16;11:1113994. doi: 10.3389/fbioe.2023.1113994 (PMC9978007; doi:10.3389/fbioe.2023.1113994)

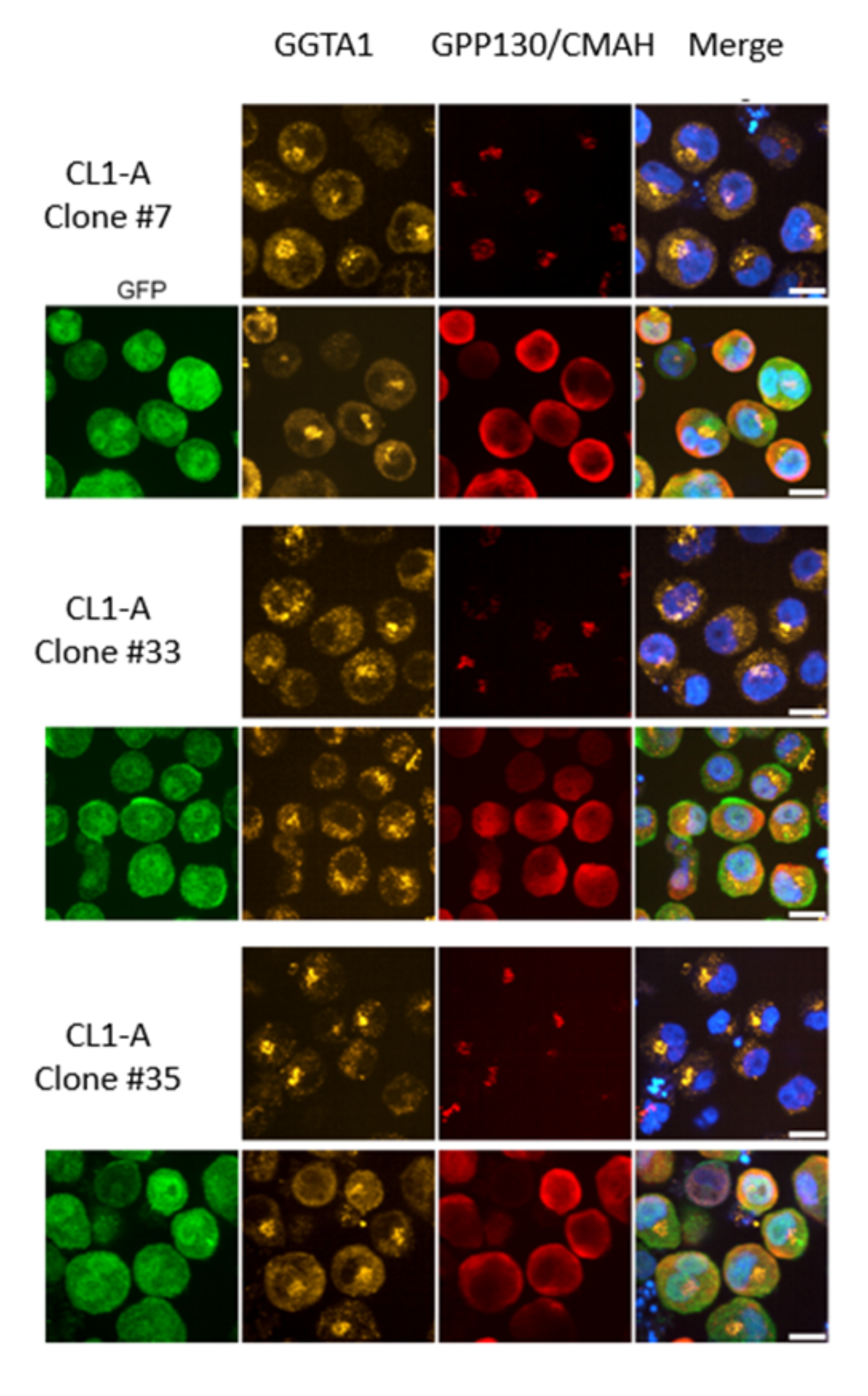

Supplement: Supplementary file 1 [file Image1.TIF]
